# Developing the DIGIFOOD Dashboard to Monitor the Digitalization of Local Food Environments: Interdisciplinary Approach

**DOI:** 10.2196/59924

**Published:** 2024-08-13

**Authors:** Si Si Jia, Xinwei Luo, Alice Anne Gibson, Stephanie Ruth Partridge

**Affiliations:** 1 Susan Wakil School of Nursing and Midwifery Faculty of Medicine and Health University of Sydney Sydney Australia; 2 Sydney Informatics Hub University of Sydney Sydney Australia; 3 Menzies Centre for Health Policy and Economics Faculty of Medicine and Health University of Sydney Sydney Australia

**Keywords:** online food delivery, food environment, dashboard, web scraping, big data, surveillance, monitoring, prevention, food, food delivery, development study, development, accessibility, Australia, monitoring tool, tool, tools

## Abstract

**Background:**

Online food delivery services (OFDS) enable individuals to conveniently access foods from any deliverable location. The increased accessibility to foods may have implications on the consumption of healthful or unhealthful foods. Concerningly, previous research suggests that OFDS offer an abundance of energy-dense and nutrient-poor foods, which are heavily promoted through deals or discounts.

**Objective:**

In this paper, we describe the development of the DIGIFOOD dashboard to monitor the digitalization of local food environments in New South Wales, Australia, resulting from the proliferation of OFDS.

**Methods:**

Together with a team of data scientists, we designed a purpose-built dashboard using Microsoft Power BI. The development process involved three main stages: (1) data acquisition of food outlets via web scraping, (2) data cleaning and processing, and (3) visualization of food outlets on the dashboard. We also describe the categorization process of food outlets to characterize the healthfulness of local, online, and hybrid food environments. These categories included takeaway franchises, independent takeaways, independent restaurants and cafes, supermarkets or groceries, bakeries, alcohol retailers, convenience stores, and sandwich or salad shops.

**Results:**

To date, the DIGIFOOD dashboard has mapped 36,967 unique local food outlets (locally accessible and scraped from Google Maps) and 16,158 unique online food outlets (accessible online and scraped from Uber Eats) across New South Wales, Australia. In 2023, the market-leading OFDS operated in 1061 unique suburbs or localities in New South Wales. The Sydney-Parramatta region, a major urban area in New South Wales accounting for 28 postcodes, recorded the highest number of online food outlets (n=4221). In contrast, the Far West and Orana region, a rural area in New South Wales with only 2 postcodes, recorded the lowest number of food outlets accessible online (n=7). Urban areas appeared to have the greatest increase in total food outlets accessible via online food delivery. In both local and online food environments, it was evident that independent restaurants and cafes comprised the largest proportion of food outlets at 47.2% (17,437/36,967) and 51.8% (8369/16,158), respectively. However, compared to local food environments, the online food environment has relatively more takeaway franchises (2734/16,158, 16.9% compared to 3273/36,967, 8.9%) and independent takeaway outlets (2416/16,158, 14.9% compared to 4026/36,967, 10.9%).

**Conclusions:**

The DIGIFOOD dashboard leverages the current rich data landscape to display and contrast the availability and healthfulness of food outlets that are locally accessible versus accessible online. The DIGIFOOD dashboard can be a useful monitoring tool for the evolving digital food environment at a regional scale and has the potential to be scaled up at a national level. Future iterations of the dashboard, including data from additional prominent OFDS, can be used by policy makers to identify high-priority areas with limited access to healthful foods both online and locally.

## Introduction

### Background

Food environments are shaped by physical, economic, political, and sociocultural factors, which in turn can determine the accessibility, availability, and affordability of foods [1]. Food environments are therefore considered powerful influences of dietary behaviors and health. Two main food environments have been described by Cummins and Macintyre [2]—one in which individuals shop for food intended for home consumption—from grocery stores and supermarkets, and another where foods are purchased for out-of-home food consumption—from restaurants and takeaways.

The World Health Organization (WHO) [3] has reported exponential growth of the out-of-home food sector, and evidence suggests that out-of-home foods are higher in energy, saturated fats, sugar, and salt, which may elevate the risk for obesity and associated diet-related chronic diseases such as type 2 diabetes and cardiovascular disease. Despite this, research on the impact of food environments on dietary and health outcomes remains conflicting. An umbrella review of 32 reviews found evidence of an association between fast-food exposure and greater body weight, although the risk of bias for these studies was high [4]. Another systematic review of 15 studies showed there was no association between availability and distance to healthy food outlets and on dietary intake and BMI in adults [5]. In children aged up to 12 years, however, there was a significant association between healthy food access and better dietary intake and lower BMI [5].

Adding to these complex relationships, in recent years, digital technology has also transformed the accessibility and availability of foods. In the current Information Age, digital technologies are deeply integrated into all areas of life and have transformed the way people work, communicate, and consume services and goods. As a result, rapid digitalization has also transformed out-of-home food environments, leading to the rise of online food delivery services (OFDS). These platforms are central agents in the digitalized food system where individuals interact with information and services from online settings to access and purchase foods [6]. The global OFDS market size is expected to reach over US $1.2 trillion in 2024 and has infiltrated multiple markets worldwide including China, the United States, and the United Kingdom [7].

The resulting disintegration of geographical boundaries of food environments via delivery services may further obfuscate the complex relationship between physical food surroundings and dietary health. Research suggests that OFDS enable individuals to order food from outlets located over 3 km away [8], which is 3 times the distance of what is considered a traditional 1 km “local neighborhood environment” [1]. While increased access to a greater variety of food outlets could be beneficial for individuals and households living in areas that are highly concentrated with fast-food or other unhealthy food outlets, there are also considerable potential harms related to OFDS. Numerous studies have shown a high proportion of popular menu items offered through OFDS are high in fat, salt, and sugar [9-11] and energy dense [12]. Thus, increased access to food outlets enabled by online food delivery has the potential to further exacerbate existing obesogenic food environments. Rising consumption of out-of-home foods contributing to unhealthy diets has been observed in countries including Australia [13], China [14,15], and the United Kingdom [16].

It is therefore critical to examine existing food environments and changes resulting from the digitalization of food environments. A study in China showed that the local neighborhood food environment is a key determinant for using food delivery services [17]. Residents in China who had better access and more choices of healthy foods near their homes were less likely to order food through an OFDS. In contrast, those who worked in suburban areas were more likely to order food through delivery service compared to those working in urban areas, as urban workers had better food accessibility. This highlights an interesting relationship between food environments including both around the workplace and home and the resulting use of OFDS.

The WHO [18] has advocated for monitoring the nutritional quality of meal delivery apps to potentially inform and change policy. Nutrition surveillance is critical to informing policy planning and improving nutrition at the population level. Artificial intelligence tools can be used to collect data and merge with databases to provide a snapshot of the nutritional quality across the food landscape. A systematic review has evaluated 13 publicly available interactive data-driven dashboards as strategies for nutrition surveillance [19], including examples such as John Hopkins University’s Food Systems Dashboard [20] and the Global Food and Nutrition Security Dashboard [21]. The goals of these dashboards have focused on sharing global nutrition data, supporting evidence for nutrition programs and policies, and monitoring and tracking nutritional progress and gaps. Moreover, a review on the current landscape of nutrition informatics (nutri-informatics) [22] has recommended greater integration of nutrition into computational biomedical sciences and a need for further representation of public health nutrition investigations. In 2023, the WHO developed a novel digital platform to monitor meal delivery apps across Europe and currently has collected data from Ireland, Italy, Slovakia, Spain, and the United Kingdom [23]. The WHO platform provides important information about the digital food landscape in Europe including the most common restaurants per region, average kilojoule content per meal type, and energy value per monetary unit [23]. There is therefore indication of growing development and use of data visualizations in nutrition and health domains, which may strengthen research relating to the determinants of nutritional status to inform future policies and strategies.

### Objectives

In this study, we introduce the development of the DIGIFOOD dashboard, a data-driven tool to monitor the emerging impact of OFDS on local food environments in New South Wales, Australia. The dashboard visualizes the geographical accessibility and healthfulness of local versus online food outlets. The primary aim of this study was to describe the development process of the DIGIFOOD dashboard as a collaborative effort between public health nutrition researchers and data scientists. We present three main components to this development process: (1) data acquisition of food outlets via web scraping, (2) data cleaning and processing, and (3) visualization of food outlets on the dashboard.

## Methods

### Data Acquisition—Uber Eats Data

Web-scraping services (Scraping Solutions, a commercial provider) were used to scrape data from Uber Eats, the market-leading OFDS in Australia, to extract information on all partnering outlets located across New South Wales, which is the most populous state in Australia. Web scraping involves the creation of code or programs to automatically download and extract data from publicly available websites. This method has previously been used by public health researchers in the United Kingdom to obtain a data set of food outlets from Just Eat, a leading OFDS [24]. Data scrapers were not logged in to any personal accounts and set the scrape date and time to Friday between 6 PM and 6:30 PM to simulate when food outlets would operate for dinner. Data were scraped in 2021 for a prototype of the dashboard and again in 2023 for the current larger project.

In phase 1, we obtained data on all possible suburb and locality names that were serviced by Uber Eats across New South Wales, Australia, including suburb or locality name, state or territory, postcode, longitude, and latitude. Using the geographical coordinates, we obtained the midpoint of the search suburb or rural locality name. In January 2023, the Uber Eats Australia website listed 52 locations across New South Wales where delivery was available (see Table S1 in Multimedia Appendix 1). We matched all locations listed on the Uber Eats website to a possible total of 1828 suburb and locality names (out of a total of 4524 individual names across New South Wales using data from Australia Post). The 1828 suburb and locality names and postcodes were provided to web scraping as input to search all Uber Eats food outlets in New South Wales. Of these suburb and locality names, 1061 returned search results from Uber Eats, and publicly available data were subsequently obtained in 3 phases. In phase 2, all suburbs from phase 1 were searched in Uber Eats, and data were extracted from all resulting food outlets that delivered to the searched suburb or locality name. Food outlet data included the name of the food outlet, street address, suburb, state, postcode, opening and closing hours, rating of food outlet, categories, delivery fee, latitude and longitude, and restaurant ID. In phase 3, geographical distances from food outlets to the midpoint of the suburb or locality name identified from phase 1 were obtained (n=66,948). Table S2 in Multimedia Appendix 1 presents an explanation of these terms.

### Data Acquisition—Google Maps Data

Google Maps was used as a source of data to represent the local food environment (labeled as “local” food outlets). These food outlets may be inclusive of those that are on Uber Eats and represent the brick-and-mortar location. According to market research, 93% of consumers use Google Maps to search for local stores and businesses [25]. Google consumer insights also revealed that 89% of dining research is done by mobile before visiting a restaurant [26]. Moreover, several public health studies have found Google data to be a reliable source for identifying food retailers [27-30] comparable to in-person ground-truthing audits. Thus, it is likely that most food outlets have a Google Maps business listing and can be representative of the local food environment.

Relevant search terms (n=120) representative of the food environment were obtained from a list of over 4000 Google My Business categories, publicly available online [31]. These were search terms containing keywords that study investigators considered had aligned with predominant food outlets described in the food environment literature including cafes, convenience stores, restaurants, supermarkets, and takeaway [32,33]. Table S3 in Multimedia Appendix 1 present the full list of Google search terms.

Next, each of these 30 terms was searched in all suburb and locality names serviced by Uber Eats as obtained in phase 2 of the Uber Eats data scrape (n=1061). Using a Google application programming interface (API) key, relevant data from food outlets on Google Cloud platform services were scraped. Briefly, an API is an intermediary between websites and software where data and information are exchanged when requests are sent [34]. The Google Places API responds to HTTP queries with information about locations. This API defines places as establishments, geographic locations, and notable points of interest [35]. The Google Places API’s nearby search function delivers a list of all instances of the specified keyword within a fixed radius (eg, “Italian Restaurant in Leichhardt NSW 2040, Australia”). The following data were collected: input query, name, review rating, number of reviews, price category, category, service options, address 1, suburb, state, postcode, opening hours, latitude, longitude, food outlet, URL, RefURL, and scrape date.

### The Local, Online, and Hybrid Food Environments

**Figure 1:**
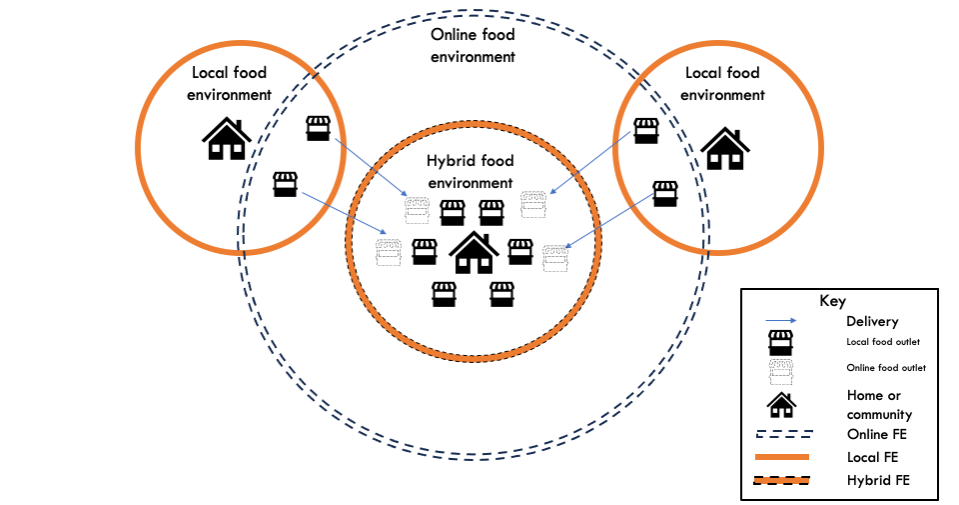
Conceptual diagram of the online food environment versus local food environment. Food outlets of the local food environment are geographically bound by a physical distance—in our study, this was defined using postcode. To observe the impact of online food delivery services on local food environments, we conceptualized the “hybrid food environment” as the combination of local and online food outlets minus the overlapping outlets. In this figure, the hybrid food environment would be the local food environment with the extra food outlets from the online food environment that can deliver in. FE: food environment.

**Figure 2:**
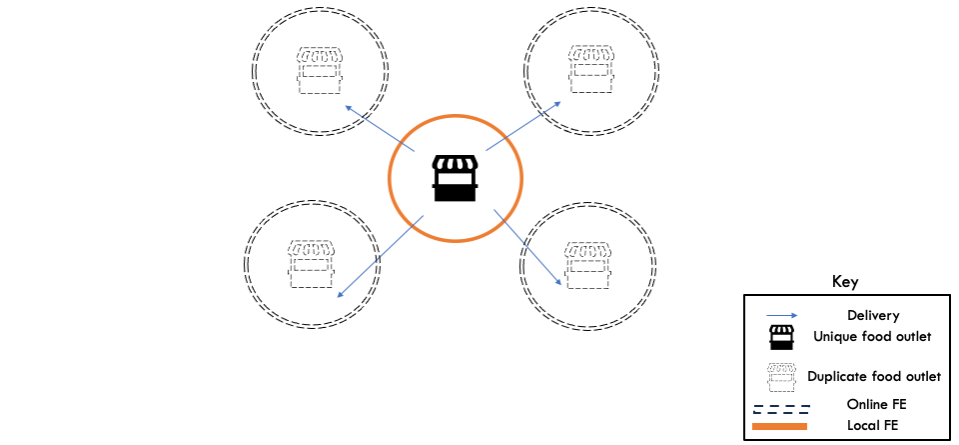
Unique food outlets on Uber Eats were counted as those that had a physical brick-and-mortar store location that offered delivery to neighboring postcodes. Duplicate outlets were counted as all the various locations where the unique food outlet delivered to via online food delivery services. FE: food environment.

Data from Uber Eats were used as measures of the online or digital food environment, while data from Google Maps were used as measures of the local or physical food environment. The hybrid food environment was defined as the collective online and local food environment to account for the impact of OFDS. Figures 1 and 2 provide a conceptual diagram of how we have defined these food environments using the data we have scraped.

### Data Cleaning

#### Identifying Unique Outlets on Uber Eats (Online Food Environment)

Following the 3 phases of data scraping from Uber Eats, a full set of data was obtained. After removing duplicate food outlets (ie, food outlets that appeared more than once in a delivery suburb where the outlet delivered to), as opposed to the physical suburb (where the outlet was located), this resulted in 16,158 unique food outlets. These were identified using the unique ID linked to each food outlet on Uber Eats.

#### Identifying Unique Outlets on Google Maps (Local Food Environment)

A full data set of 64,239 food outlets from Google Maps was obtained on July 18, 2023. Irrelevant nonfood businesses, for example, appliance store, corporate office, and jewelry store, were filtered out. In addition, outlets that were tagged as “permanently closed” were also filtered out. As such, this was further refined to 36,967 unique food outlets. Unique Google Maps outlets were identified using their Google Customer ID, which Google assigns to an individual business entity.

### Matching Outlets From Uber Eats and Google Maps Data

#### Overview

It was anticipated that the Uber Eats data would be a subset of the Google Maps data since most online food outlets on Uber Eats would have a physical store location typically listed on Google Maps as a business. Thus, we expected to see nearly all Uber Eats outlets (n=16,158) to have matched to an outlet in the Google Maps data set (n=36,967). To avoid duplication of food outlets from the 2 data sets, common food outlets (ie, outlets that were both listed on Google Maps and offered delivery service via Uber Eats) were matched by names and addresses. Only relevant columns from both the Google Maps data set and the Uber Eats data were kept for matching including suburb, street address, postcode, state, name, CID, and category.

#### Processing Data for Matching

##### Suburb

In the “suburb” column, all string data were converted into lowercase, special characters such as a dash or quote were removed, and spaces were kept in. Any suburbs that were not part of New South Wales were removed, for example, suburbs in the Australian Capital Territory were excluded.

##### Street Address

Similarly, in the “address 1” column, all string data were converted into lowercase. If the street address also contained the suburb name, postcode, and state, these were removed such that only the address component was kept. If food outlets were located in shopping centers, multilevel buildings, central areas, or plazas, this part of the name was removed. Multimedia Appendix 1 presents the list of manually identified food courts, centers, and buildings.

##### Name of Outlet

All string data of food outlet names were converted into lowercase, and all special characters such as apostrophes and nonalphanumerical characters were removed. If the name of the food outlet had included the name of food centers, petrol station, suburb, city (Sydney), or state (New South Wales), this would be subsequently removed from the name.

##### Missing Information

Mandatory fields were required for the parsing process, which converts the data from one format into another format. In cases where there were postcodes available and suburb names were missing, a suburb name that was linked most to the same postcode from other food outlets would be used instead. For example, the postcode “2000” most commonly had the suburb name “Sydney,” although a small proportion could also be “Haymarket”—as an assumption, Sydney would be used instead of Haymarket to fill in the empty suburb name. If both the suburb name and postcode were missing, then the fields would be filled in with “empty” and “0000,” respectively. If the street address had no street number, it would be filled in with “12345” in front.

#### Matching Criteria

Food outlets were matched using the processed names and addresses of food outlets in both Uber Eats and Google data sets. A combination of the following various matches was used. With each step, fewer restrictions were placed on the matching criteria. Table 1 provides the sequence in which food outlets were matched based on differing criteria.

**Table 1:** Sequence of criteria for matching food outlets between Uber Eats and Google Maps data sets.

Step	Criteria for matching
1	Same name, street name, suburb, postcode
2	Same name, street name, suburb
3	Same name, street name, postcode
4	Same name, postcode
5	Same name, suburb
6	Same name, street name, street type
7	Same name, suburb, postcode
8	Same name, street number, street name
9	Same name, street number, suburb, postcode
10	Same street number, street name, suburb, postcode
11	Same street name, suburb, postcode
12	Same postcode
13	Same suburb
14	Same street name
15	Same street type

For steps 10 and 11, outlets were considered a match if they had fulfilled the criteria and if the names met the 83% similarity threshold. This threshold level was chosen from observation after trial and error. Food outlets were matched using “jelly” (similarity scale from 0 to 1) and “fuzzy” (similarity scale from 0 to 100) matching algorithms if their similarity scores for both name and address were above 90%.

The resulting 2000 unmatched outlets were manually verified. Reasons for errors in matching included inconsistencies in addresses when food outlets were located within multilevel buildings (eg, shopping centers such as Westfield and Central Park) or central food areas (eg, Spice Alley), incorrectly formatted addresses (eg, state is in the postcode column or postcode is in the state column), naming inconsistencies for suburbs (eg, “Brighton Le Sands” and “Brightonlesands”), naming inconsistencies for food outlet names, and incomplete scrape of food outlet data from Google Maps. After accounting for the reasons, 66% (10,708/16,158) of outlets were matched between the 2 data sets (Uber Eats and Google).

### Categorization of Food Outlets

#### Overview

To descriptively analyze the healthfulness of local and online food environments, we assigned food outlets 1 of 9 categories based on the Food Environment Score, which was developed by a group of Australian public health and nutrition experts in a modified Delphi survey [36]. The 9 main categories included takeaway franchises, independent takeaways, independent restaurants or cafes, supermarkets, fresh produce, sandwich shops or salad bars, sweets or extra foods, bakeries, and convenience stores or service stations. Table S2 in Multimedia Appendix 1 presents the definitions of these categories.

**Figure 3:**
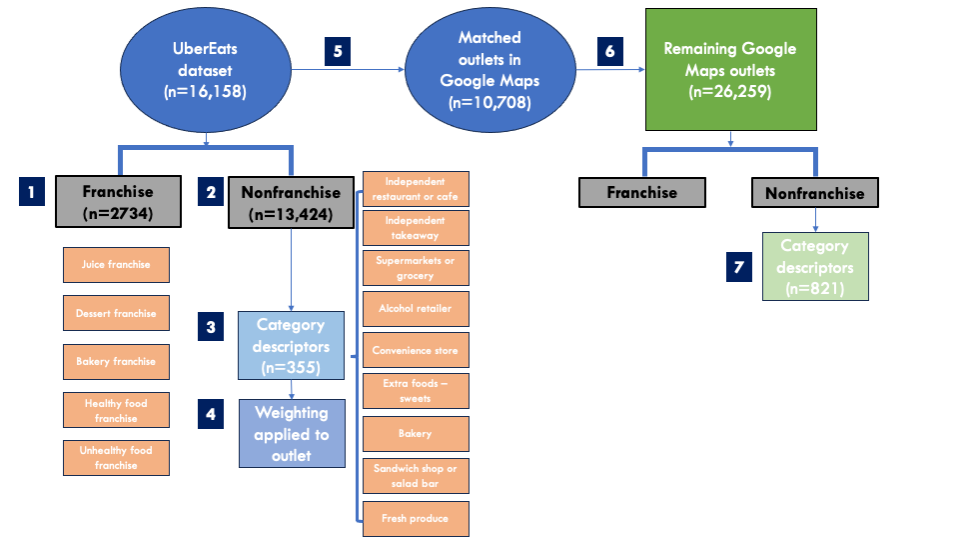
Categorization process of food outlets. Step 1: Outlets were first assigned as franchise or nonfranchise. Steps 2-3: 1 of 9 main categories was assigned to each category descriptor. Step 4: The predominant main category for each food outlet was based on the weighting of various category descriptors. Steps 5-7: The same process was repeated for food outlets from the Google Maps data set.

Initially, outlets were labeled either as a franchise or nonfranchise outlet (step 1 in Figure 3). We defined a franchise as a brand that has more than 10 different locations in the data sets. Under menu labeling legislation in the state of New South Wales, franchises or “standard food outlets” are defined as having greater than 20 locations in the state or over 50 nationally. We used a more generous definition for franchise, to be inclusive rather than exclusive, accounting for any potential missing data that would result in a true franchise store incorrectly assigned to the nonfranchise category.

#### Nonfranchise Outlets

For nonfranchise outlets, we first manually assigned a main category to all category descriptors in the Uber Eats data set (n=355). For example, a category descriptor of “Asian” would be assigned a main category of independent restaurant or cafe. Some category descriptors were relevant to more than 1 main category. These cases included “Grocer,” which was assigned both supermarkets and fresh produce; “Hotel,” which was assigned alcohol retailer and independent restaurant or cafe; and “Restaurant,” which was relevant for independent restaurant or cafe and independent takeaway. If there were any irrelevant non–food-related category descriptors that could not be assigned a main category, they were assigned “N/A” (steps 2 and 3 in Figure 3).

All these category descriptors and their assigned main category received a base score of 2. Using the previous example, “Asian” received a score of 2 for independent restaurant or cafe, while “Chicken” received a score of 2 for independent takeaway. Weightings for certain categories were tweaked to adjust for the prominence; for example, “Fast food” would receive a score of 4 for independent takeaway, while specific and more niche category descriptors such as “Japanese set item” would receive a score of 1 for independent restaurant or cafe (step 4 in Figure 3).

In majority of cases where food outlets had more than 1 category descriptor, the main category would be assigned based on the highest cumulative score. Using “Shawarma Today” as an example, the associated category descriptors were comfort food, burger, and salad. Comfort food received a score of 2 for independent takeaway, burger received a score of 2 for independent takeaway, and salad received a score of 2 for sandwich shop or salad bar. Tallying up the scores, independent takeaway received a total score of 4, while sandwich shop or salad bar only received a score of 2. Hence overall, “Shawarma Today” was assigned independent takeaway as its main category. When the score for main categories was a tie, the main category that appeared less frequently in the total food outlet data set was assigned higher priority. This was to adjust for overrepresentation of food outlet categories and increase the diversity of different food outlet types in the data set.

The main category was transferred from the outlet in Uber Eats data set to the same matching outlet in the Google Maps data set (step 5 in Figure 3). For the remaining outlets in the Google Maps data set, a similar process was conducted using category tags extracted from Google Maps (n=821; steps 6 and 7 in Figure 3).

#### Franchise Outlets

Franchise outlets were further categorized into dessert franchise, bakery franchise, juice franchise, healthy food franchise, or unhealthy food franchise (step 1 in Figure 3). These were manually determined based on the predominant offerings of menu items. For example, franchise stores that were mainly offering sweets, cakes, ice cream, or sugar-sweetened beverages were categorized as dessert franchise. Bakery franchises were those that offered breads, pastries, and other baked goods. Juice franchises sold mainly juices and smoothies from fruits and vegetables. Healthy food franchises were assigned to stores that mostly offered salads, sushi, or sandwiches. Unhealthy food franchises were renowned fast-food chains predominantly selling burgers, pizza, and fried chicken.

### Implementation of Data Inputs Into DIGIFOOD Dashboard

Microsoft Power BI was used to geographically map and visualize local and online food outlets across New South Wales, Australia. Data files on postcodes, regions, and suburbs and localities were downloaded from the Australian Bureau of Statistics—Australian Statistical Geography Standard Edition 3 (Postal Areas 2021, Suburbs and Localities 2021, Statistical Area Level 4 2021). These files were linked by Mesh Blocks, which are the smallest geographic areas and form the building blocks for the larger regions of the Australian Statistical Geography Standard. Suburbs and localities are an Australian Bureau of Statistics Mesh Block approximation of the officially recognized boundaries of suburbs—in cities and larger towns, and localities—outside cities and larger towns, as defined by the state and territory governments of Australia. Rural or urban definitions depend on the density or urban infrastructure criteria. Population numbers for each region in New South Wales were obtained from the 2021 census data [37]. Table S2 in Multimedia Appendix 1 presents further details.

The 3 main components of the dashboard included a geographical map of online, local, and hybrid food outlets; a pie chart of the main categories of food outlets; and a word cloud that visualizes the frequency of cuisines across a specified area.

### Statistical Analysis

Descriptive statistics were used to analyze data in the DIGIFOOD dashboard, and the results are presented in this manuscript.

### Ethical Considerations

Ethics approval was not required. This work does not contain any data involving human participants. Data on food outlets obtained via web scraping were publicly available and used for noncommercial research purposes.

## Results

### Data Overview

Of the 4524 suburb and locality names across New South Wales, 1061 were serviced by Uber Eats at the time of data acquisition. To date, 36,967 unique food outlets representing the local food environment and 16,158 unique food outlets representing the online food environment in New South Wales, Australia, have been mapped on the DIGIFOOD dashboard. These figures potentially suggest that 43.7% (16,158/36,967) of all available food outlets are accessible via the market-leading OFDS in Australia.

The highest number of online food outlets (n=4221) and locally accessible food outlets were found in the Sydney-Parramatta region (n=10,069), which encompasses 28 postcodes in the scraped data set. In contrast, the Far West and Orana region, which covers 2 postcodes, had the lowest number of locally accessible food outlets (n=7; Table 2).

**Table 2:** Number of local, online, and hybrid outlets by regions in New South Wales, Australia, ordered by ascending number of local outlets.

New South Wales region	Postcodes, n	2021 census population, n	Local outlets, n	Online outlets, n	Hybrid outlets^a^, n	Change in local food outlets resulting from OFDS^b,c^, n/N (%)
Far West and Orana	2	115,566	299	7	300	299/300 (100)
Coffs Harbour-Grafton	4	146,127	667	7	667	667/667 (100)
New England and North West	4	185,560	893	25	905	893/905 (101)
Southern Highlands and Shoalhaven	2	161,006	1031	3	1031	1031/1031 (100)
Murray	3	123,552	1175	159	1219	1175/1219 (104)
Richmond-Tweed	8	257,061	1317	97	1352	1317/1352 (103)
Capital Region	5	238,810	1345	57	1374	1345/1374 (102)
Mid North Coast	7	229,035	1559	20	1564	1559/1564 (100)
Riverina	10	161,349	1562	164	1634	1562/1634 (105)
Central West	6	212,962	1776	16	1778	1776/1778 (100)
Central Coast	10	346,596	4860	339	5059	4860/5059 (104)
Newcastle and Lake Macquarie	21	390,519	5232	297	5350	5232/5350 (111)
Hunter Valley exc Newcastle	12	291,946	5036	686	5569	5036/5569 (102)
Sydney-Sutherland	11	229,604	5611	514	6022	5611/6022 (107)
Illawarra	16	313,842	5626	824	6081	5626/6081 (108)
Sydney-Ryde	11	202,038	6179	1309	7302	6179/7302 (118)
Sydney-Northern Beaches	20	263,554	6190	839	6930	6190/6930 (112)
Sydney-Outer South West	14	297,292	6319	1077	7148	6319/7148 (113)
Sydney-Eastern Suburbs	16	261,410	6618	2170	8604	6618/8604 (130)
Sydney-Blacktown	16	396,776	7029	2035	8683	7029/8683 (124)
Sydney-North Sydney and Hornsby	26	424,045	7046	1728	8633	7046/8633 (123)
Sydney-Baulkham Hills and Hawkesbury	14	264,371	7515	1592	8709	7515/8709 (116)
Sydney-Inner West	25	304,771	8070	3562	11,228	8070/11,228 (139)
Sydney-Outer West and Blue Mountains	24	332,056	8130	1609	9128	8130/9128 (112)
Sydney-City and Inner South	26	331,340	8684	3850	12,282	8684/12,282 (141)
Sydney-South West	21	474,430	8978	2462	10,808	8978/10,808 (120)
Sydney-Inner South West	31	605,118	9464	3231	12,335	9464/12,335 (130)
Sydney-Parramatta	28	493,515	10,069	4221	13,758	10,069/13,758 (137)
Total			36,967	16,158	51,470	36,967/51,470 (139)

^a^Hybrid food outlets represent the combined local and online food outlets within the region. Figure 1 and Table S2 in Multimedia Appendix 1 provides further information.

^b^OFDS: online food delivery services.

^c^Percentage change in food outlets resulting from OFDS was calculated as the number of hybrid outlets divided by the number of local outlets, multiplied by 100.

Regions where OFDS had the most significant impact were Sydney-City and Inner South, Sydney-Inner West, Sydney-Parramatta, Sydney-Eastern Suburbs, and Sydney-Inner South West in descending order. These regions all observed a greater than 130% increase in total food outlets by comparing hybrid outlets to local outlets (Table 2).

In contrast, OFDS appeared to have the least significant impact in Far West and Orana, Coffs Harbour-Grafton, Southern Highlands and Shoalhaven, Mid North Coast, and Central West regions (Table 2). These regions did not observe an overall change in total food outlets, as the number of hybrid food outlets reflected 100% of the number of local food outlets (Table 2).

In both local food environments and online food environments, it was evident that independent restaurants and cafes comprised the largest proportion of food outlets at 47.2% (17,437/36,967) and 51.8% (8369/16,158), respectively (Table 3). However, compared to local food environments, the online food environment has relatively more takeaway franchises (2734/16,158, 16.9% compared to 3273/36,967, 8.9%) and independent takeaway outlets (2416/16,158, 14.9% compared to 4026/36,967, 10.9%). Noticeably, there were relatively more unhealthy food franchises (1612/16,158, 10% compared to 1961/36,967, 5.3%) and dessert franchises (706/16,158, 4.4% compared to 800/36,967, 2.2%).

**Table 3:** Categorization of food outlets accessible locally and online across New South Wales, Australia.

Categories		Local (n=36,967), n (%)^a^		Online (n=16,158), n (%)^a^	Hybrid (n=51,470), n (%)		Local outlets that are accessible online by category, n/N (%)
Independent restaurants or cafes		17,437 (47.2)		8369 (51.8)	25,566 (49.7)		8369/17,437 (48)
Independent takeaway		4026 (10.9)		2416 (14.9)	6393 (12.4)		2416/4026 (60)
**Takeaway franchise**		3273 (8.9)		2734 (16.9)	5510 (10.7)		2734/3273 (83.5)
	Unhealthy food franchise	1961 (5.3)	1612 (10)		3200 (6.2)	1612/1961 (82.2)	
	Healthy food franchise	213 (0.6)	188 (1.2)		387 (0.8)	188/213 (88.2)	
	Bakery franchise	254 (0.7)	131 (0.8)		373 (0.7)	131/254 (51.6)	
	Dessert franchise	800 (2.2)	706 (4.4)		1411 (2.7)	706/800 (88.3)	
	Juice franchise	45 (0.1)	97 (0.6)		139 (0.3)	97/45 (216)	
Supermarkets or grocers		2441 (6.6)		217 (1.3)	2646 (5.1)		217/2441 (8.9)
Alcohol retailer		2242 (6.1)		590 (3.6)	2824 (5.5)		590/2242 (26.3)
Convenience stores or petrol station		2117 (5.7)		541 (3.3)	2632 (5.1)		541/2117 (25.6)
Fresh produce (excluding groceries)		2106 (5.7)		44 (0.3)	2149 (4.2)		44/2106 (2.1)
Extra foods—sweets		1613 (4.4)		678 (4.2)	2260 (4.4)		678/1613 (42)
Bakery		1200 (3.2)		115 (0.7)	1307 (2.5)		115/1200 (9.6)
Sandwich shops or salad bars		512 (1.4)		454 (2.8)	950 (1.8)		454/512 (88.7)
Total		36,967		16,158	51,470		16,158/36,967 (43.7)

^a^Local and online contain duplicates between the 2 data sets.

Furthermore, Table 3 shows that most takeaway food franchises, including unhealthy food franchises, healthy food franchises, dessert franchises, and juice franchises, in addition to sandwich shops and salad bars, are offered and accessible online via the market-leading meal delivery app, as these represented close to or more than 80% of locally available food outlets. In contrast, supermarkets and grocers, fresh produce, and bakeries are the least accessible online via Uber Eats, as these represented less than 10% of the total local food outlets.

### Case Study for Application of the DIGIFOOD Dashboard: Sydney-Parramatta Metropolitan Region

#### Overview

Policy makers, especially those in local governments, could use the DIGIFOOD dashboard tool to gain insight into the local, online, and hybrid food environments in their areas of interest. This could help inform planning decisions and approval of development proposals to optimize the health and well-being of communities.

**Figure 4:**
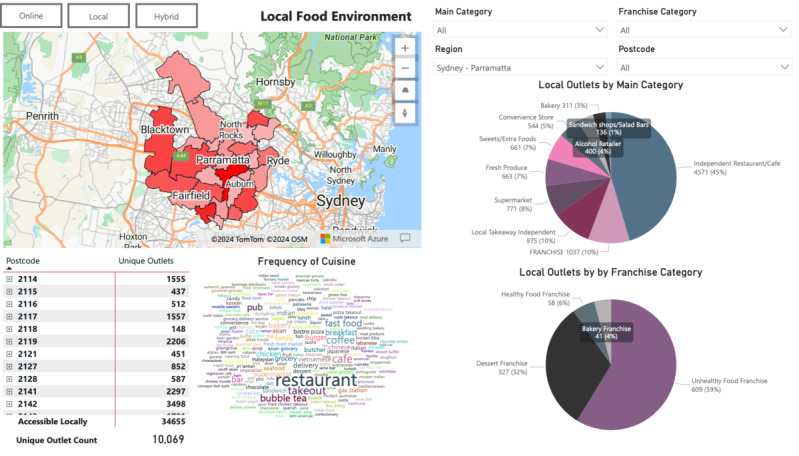
Local food environment display for the Sydney-Parramatta region.

**Figure 5:**
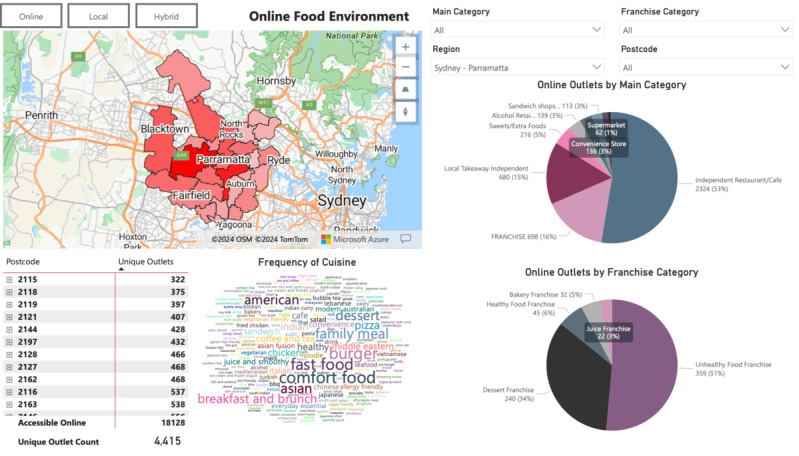
Online food environment display for the Sydney-Parramatta region.

**Figure 6:**
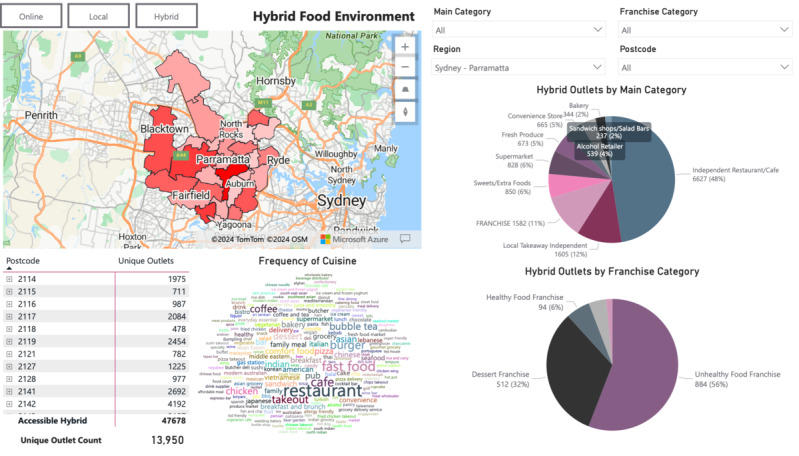
Hybrid food environment display for the Sydney-Parramatta region.

For example, the Sydney-Parramatta region of New South Wales is a major metropolitan area with a population of over 493,515 (2021 census) [38]. The dashboard also allows users to navigate between the displays of data to observe (1) local food environments, (2) online food environments, and (3) hybrid food environments. These displays are shown in Figures 4-6 using the example of the Sydney-Parramatta region. Features of the DIGIFOOD dashboard are explained in Table 4.

**Table 4:** Features of the DIGIFOOD dashboard.

Feature	Explanation	Case study example
Pie chart of food outlet categories	The pie chart represents the composition of food outlets by main categories and franchises.	In Figure 5, the pie chart shows that online food outlets were mostly comprised of unhealthy food franchises, followed by dessert franchises, healthy food franchises, and bakery franchises.
Frequency of cuisine word cloud	The word cloud displays the frequency of “cuisines,” also known as category descriptors for food outlets. Larger words represent a higher frequency of the cuisine.	In Figure 6, the word cloud shows a large variety of cuisines available in the hybrid food environment. Cuisines that were more prominent included restaurants, fast food, burgers, and bubble tea.
Map density	In the map section, areas that were of a darker red color indicated a higher relative density of food outlets sorted by postcode.	In Figures 4-6, the postcode 2148 for Blacktown in the Sydney-Parramatta region appears to have a high density of local, online, and hybrid food outlets as determined by the darker red color.
Search by postcode	In addition to searching food environments by region, the dashboard enables a search by postcodes of interest. This will then provide data specific to the postcode, including frequency of cuisine word cloud and pie charts of the categorization of food outlets.	For instance, users can obtain a closer look at postcode 2148 to observe changes in the frequency of cuisine, composition of categories, and number of food outlets.

#### Comparison of the 3 Different Food Environments

At the regional level, it was clear that compared to the local food environment, the online food environment was offering more franchise stores—with a higher proportion of juice and bakery franchises available, as shown in the pie charts (Figure 4). From the word cloud display in Figure 5, it was clear that the online food outlets were comprised mainly of fast food, comfort food, family meals, and American food. Figure 6 shows the significant increase in a number of food outlets in the hybrid food environment compared to the local food environment. The pie charts in Figure 6 compared to Figure 4 also demonstrated substantial growth in unhealthy food franchises.

## Discussion

### Principal Findings

Nutrition surveillance is fundamental to informing healthy policy planning at the population level [6,39]. OFDS are expanding food environments and increasing food accessibility within local neighborhoods or communities. The changing access to more healthful or unhealthful food outlets has important implications on population health and nutrition. Monitoring the digitalization of food environments is therefore essential for guiding policy development to ultimately enhance nutritional outcomes across the population.

This study presents the development of the DIGIFOOD dashboard to monitor changes in the local food environments of New South Wales in Australia. We explain our multidisciplinary approach to developing the DIGIFOOD dashboard to monitor the availability and accessibility of both local and online food outlets. This tool can also identify areas most affected by adverse changes resulting from the digitalization of food environments, where healthy food options are outnumbered by unhealthy food options.

The WHO Europe has similarly developed a dashboard to monitor the out-of-home food environment [23]. The platform currently collects data from 2 major food delivery platforms in Europe and provides information on available restaurants, their geographical location and delivery reach, as well as menu items if available [23]. Where the energy information of menu items is provided, the WHO dashboard developed by Hetz et al [23] can provide a comparative analysis of average calories per restaurant or restaurant type. However, it was noted by Hetz et al [23] that a lack of data on menu items with energy labeling can limit such analyses. Furthermore, recent studies have shown that menu labeling is largely absent and inconsistent on OFDS [40-42], and only franchise restaurants are mandated to provide nutritional labeling. The DIGIFOOD dashboard shows that nearly 50% of food outlets on OFDS are smaller independent takeaways and independent restaurants and cafes. These outlets therefore may not have the resources to calculate or provide nutritional information of menu items.

### Digitalization Is Changing the Geographical Boundaries of Food Environments

The DIGIFOOD dashboard provides unique insight into the impact of digitalization on the geographical boundaries of traditional local food environments. We demonstrate that in New South Wales, the overall hybrid food environment that accounts for the collective impact of online and local food outlets is largely comprised of independent restaurants or cafes (25,566/51,470, 49.7%), followed by independent takeaway outlets (6393/51,470, 12.4%) and franchise takeaway outlets (5510/51,470, 10.7%). These findings suggest that indeed local food environments are becoming further saturated by additional food outlets accessible online and can get delivered in.

This study also showed that the leading OFDS in Australia appeared to have the greatest impact in metropolitan urban areas compared to the outer regional and rural areas. While regions close to the central business district in Sydney were observed to have a greater than 130% increase in total food outlets from food outlets accessible via delivery, regions in rural areas with a population size 3 to 4 times smaller observed little to no change to total food outlets. It is important to note that, however, these observations have been derived from only 1 OFDS, and it is likely that other competitors may have more prominence in these regional areas.

Another study conducted in China showed that the density of takeaway food facilities offering OFDS was also more concentrated in urban central areas [43]. In contrast, compared to more central urban areas, peripheral areas observed a decreasing trend in the density of these online food outlets. Moreover, researchers in Nanjing, China, established a spatial correlation between online-to-offline food delivery restaurants and fast-food restaurants that provide meals high in fat, oil, and salt [44]. This finding suggests that compared to conventional restaurants that only offer dine-in service restaurants offering online food delivery are more geographically connected to fast-food restaurants (Pearson coefficient of 0.42 and *q* value of 0.79; *P*<.001). Hence, online food delivery may further promote accessibility to fast-food restaurants and increase consumption of fast food.

Janatabadi et al [45] have also investigated the social and spatial inequalities of contemporary food desserts in the United Kingdom. A positive association between physical and online access to groceries and health conditions was found [45]. Among individuals with poor health conditions, roughly 550,000 had relatively higher access to online grocery services accompanied by a relatively low level of physical store access [45]. As health conditions improved, the reliance on online food shopping was shown to decrease, and those individuals had higher access to physical grocery stores [45]. This finding potentially suggests that increasing access to OFDS for groceries may be an important consideration for those living in more deprived areas who may face more health challenges or poorer health conditions. Altogether, it is evident that digitalization may be changing the geographical or spatial boundaries of food outlet access. Thus, it is of key interest to continue examining the potential impacts of digital food environments on population health.

### Web Scraping

Similar to this study, research from Great Britain used scraped data from food outlets available on Just Eat, the market-leading OFDS in the United Kingdom, as the training material. Web scraping enables easy access to large volumes of data and can help overcome knowledge gaps [46]. In recent years, public health researchers have increasingly used web-scraping techniques to obtain data for analyses; for example, in Canada, researchers obtained food price data from the websites of the largest food retailer corporation, Loblaws [47], to perform food cost analyses. Online food delivery platforms uniquely offer a wealth of publicly available data that can be used to investigate out-of-home food consumption as demonstrated in previous studies [48,49]. Collaboration between data scientists and public health nutrition researchers to use and analyze big data is essential for further research in the field of digital food environments.

### Limitations

While web scraping is generally a more efficient way to obtain large volumes of data from online sources, there were several limitations with the quality of data we scraped. We obtained Google Maps data by searching for various food categories of restaurants in a specified suburb within New South Wales using 30 relevant Google Business categories that were identified from a list with over 4000 categories. This therefore may not have completely captured the food environment and, moreover, was a time-intensive step, which required up to 3 months to complete due to the limits on API requests in 1 scrape [50].

In addition, we based our categorization of food outlets to characterize local, online, and hybrid food environments from a small list of food outlet categories in the Food Environment Score. These categories or outlet types were broad and resulted in nearly 50% of total food outlets categorized as independent restaurants or cafes. We attempted to adjust this overrepresentation by assigning the category that appeared less frequently whenever there was a tie between categories. We also note that there may be potential inaccuracies, as the food outlet data are often provided by business owners of food outlets. For example, it was noted that Oporto, a fast-food franchise store selling predominantly burgers and chips, had the following categories on Uber Eats: Burgers, Vegan, Desserts, Fast food, Chicken, Family Meals, and Healthy. This limitation, however, is likely to have minimal impact overall, as the main category has factored for the variety of different associated category descriptors or cuisine tags for varying outlets.

Furthermore, we only scraped data from 1 OFDS, Uber Eats, which is the market-leading platform in Australia. This may have underestimated the spread or accessibility of the “digital food environment,” as we did not account for restaurants that may have partnered with other popular food delivery services such as Menulog or DoorDash.

We also acknowledge that the data presented in the current version of the DIGIFOOD dashboard are subject to rapid changes in the opening and closing of new food outlets, in addition to growing numbers of partnering outlets with Uber Eats, especially in more rural and remote regions. This indicates that the dashboard must be continuously updated with the latest data to remain relevant and reflective of both local and online food environments.

### Future Directions

The immediate next steps for this research will be to generate a healthfulness score to conduct analyses and compare the healthfulness of local and online food environments across the socioeconomic gradient. Local governments and policy makers will also be engaged to tailor aspects or displays of the dashboard that would be most useful to them for decision-making. For example, policy makers may seek further information to guide development plans regarding access to healthy food retail for their communities. In future iterations, the dashboard can also incorporate population data such as population density, infrastructure, amenities in local communities, and walkability measures.

### Conclusions

The DIGIFOOD dashboard provides visualization of the current digital food environment driven by the proliferation of OFDS. As such, the dashboard may be a useful tool for policy makers, allowing for monitoring of the digital food environment to devise strategies that may improve accessibility to healthful foods. Further research will be conducted to analyze differences in the healthfulness of the hybrid food environments across geographical areas of varying incomes and resources, in addition to trends between regional and remote areas compared to more urbanized areas.

## Appendix

Multimedia Appendix 1 DIGIFOOD dashboard data inputs and terminology.

## Abbreviations

API: application programming interface
OFDS: online food delivery services
WHO: World Health Organization

## Data Availability

The data sets generated during this study are available from the corresponding author on request.
