# Supplementary material for: Developing the DIGIFOOD Dashboard to Monitor the Digitalization of Local Food Environments: Interdisciplinary Approach
Source: JMIR Public Health Surveill. 2024 Aug 13;10:e59924. doi: 10.2196/59924 (PMC11350305; doi:10.2196/59924)
Supplement: Multimedia Appendix 1 [file publichealth_v10i1e59924_app1.docx]

**Table S1.** UberEats Australia locations across New South Wales in 2023. Locations data obtained from UberEats website, number of suburbs/localities obtained from Australia Post.

| **NSW Local Government Area** | **Number of suburbs or localities** |
| --- | --- |
| Albury | 15 |
| Armidale | 42 |
| Ballina | 35 |
| Bathurst | 75 |
| Blue Mountains | 29 |
| Bonny Hills | 1 |
| Bowral - Mittagong | 7 |
| Byron Bay | 40 |
| Camden Haven | 19 |
| Central Coast | 151 |
| Cessnock | 56 |
| Coffs Harbour | 31 |
| Dubbo | 47 |
| Evans Head | 1 |
| Forster – Tuncurry | 2 |
| Galston | 1 |
| Gillieston Heights | 1 |
| Goulburn | 26 |
| Grafton | 1 |
| Griffith | 15 |
| Kiama | 21 |
| Kurri Kurri – Weston | 7 |
| Lake Cathie | 1 |
| Leeton | 10 |
| Leppington | 1 |
| Luddenham | 1 |
| Maitland | 46 |
| Minmi | 1 |
| Morisset – Cooranbong | 10 |
| Mulgoa | 1 |
| Murwillumbah | 3 |
| Nelson Bay - Corlette | 3 |
| Newcastle | 50 |
| Nowra – Bomaderry | 5 |
| Old Bar | 1 |
| Orange | 8 |
| Picton | 1 |
| Pitt Town | 1 |
| Port Macquarie | 87 |
| Queanbeyan | 66 |
| Raymond Terrace | 2 |
| Richmond North | 1 |
| Shoal Bay | 1 |
| Silverdale - Warragamba | 2 |
| Sydney | 765 |
| Tamworth | 69 |
| Taree | 3 |
| Thirlmere | 1 |
| Tweed Heads | 11 |
| Wagga Wagga | 58 |
| Wollongong | 65 |
| Young | 1 |
| Total | 1898 |
| Duplicates | 70 |
| **Unique** | **1828** |

**Table S2.** Terminology dictionary.

| **Terminology** | **Definition** | **Example** |
| --- | --- | --- |
| *Scraping terminology* | |  |
| Name | Name of food outlet | Rosetti’s Cafe |
| Suburb | A geographic subdivision in Australia, used mainly for address purposes for ***urban*** areas. | Burwood |
| Locality | A geographic subdivision in Australia, used mainly for address purposes for ***rural*** areas. | Borah Creek |
| State/Territory | Administrative divisions of Australia that are self-governing polities | New South Wales |
| Postcode | A four-digit number used by Australia Post to assist with mail delivery | 2134 |
| Opening hours | Opening hours of a food outlet | Monday – 10:00 AM – 10:00PM |
| Rating of food outlet | An average rating out of 5, provided by customers | 4.4 (17 ratings) |
| Category | Category descriptors of food outlets to describe the cuisine and food offered. These descriptors differed between the UberEats and Google Maps datasets. | Vietnamese, Noodle |
| Delivery Fee | Cost to deliver from food outlet | $4.49 |
| URL | The URL of a webpage that sends visitors to a specified site using a link | https://www.ubereats.com/au/store/rosettis-cafe/djF057OcSzSN52wsfJcsZQ |
| Scrape date | Date which data was scraped | 3/9/2023 11:21 |
| Restaurant ID | Unique ID for food outlets on UberEats | 84ef9e1b-4cba-4181-9e88-2e7a93f00a7e |
| CID | Unique ID for food outlets on Google Maps | 9097067965566814117 |
| *Conceptualisation of food environments terminology* | | |
| Local food outlets | Food outlets that have a physical presence. Data scraped from Google Maps. | Mario’s Pasta, Carlton, 2218 |
| Online food outlets | Food outlets that can deliver into a specified postcode.  Data scraped from Uber Eats. | McDonald’s (Hurstville) that can deliver to Carlton 2218 |
| Hybrid food environment | The collective local and online food outlets in a specified postcode. | Mario’s Pasta + McDonald’s (Hurstville) in Carlton 2218 |
| *Dashboard terminology* | |  |
| Postal Area (POA) | An approximation by the Australian Bureau of Statistics of postcodes | 2000 |
| Mesh Block | Smallest geographic area which form the building blocks for larger regions of the Australian Statistical Geography Standard. Typically includes 30-60 dwellings. | Mesh blocks are grouped into four increasingly large statistical areas. |
| Main category | Derived from the Food Environment Score, developed by public health experts to characterise the food environment. | The following categories below are examples of a main category. |
| Supermarket | Mainly sell groceries (fresh foods, canned, packaged foods, dry foods). | Woolworths |
| Sandwich shops/salad bars | Mainly sell salads, rolls, sandwiches or wraps. | Brooklyn Bridge Deli |
| Independent restaurants/cafes | A non-franchise restaurant or cafe mostly offering dine-in service, typically culture-based cuisine | Gigi’s Pizzeria |
| Takeaway Independent | A non-franchise store mostly offering foods for takeaway such as pizza, fish and chips, burgers, chicken, kebabs. | Shawarma Today |
| Fresh produce | Mainly sells fresh meat, seafood, fruit and vegetable grocers. Delicatessens have been included in this category. | LP’s Quality Meats |
| Convenience store | Mainly sell convenience foods – mostly highly- processed foods such as packaged chips, soft drinks, lollies, packaged ice cream. Includes stores at petrol stations. | City Convenience Store |
| Alcohol retailer | Mainly sell alcoholic beverages. Includes pubs and bottle shops. | Dan Murphy’s |
| Sweets/extra foods | Mainly sell sweets, desserts, pastries, cakes, bubble-tea, donuts and more. | Sweet Lu |
| Bakery | A non-franchise store that mainly offers bread and other baked products | Sonoma |
| Takeaway Franchise | An outlet brand with greater than 10 different locations in the dataset |  |
| Bakery Franchise | Franchise that primarily sells bread and baked goods | Breadtop |
| Dessert Franchise | Franchise that sells sweets, sugar-sweetened beverages, desserts | Chatime |
| Healthy Food Franchise | Franchise that sells salads, sandwiches, wraps, rolls. | Sumo Salad |
| Juice Franchise | Franchise that sells juices or smoothies. | Top Juice |
| Unhealthy Food Franchise | Franchise that sells mostly fast-foods such as burgers, pizza, fried chicken, chips. | KFC |
| Cuisine | For easier interpretation on the dashboard, cuisine represents the ‘category’ data from scraping terminology. | Vietnamese |

**Table S3.** List of Google Business Categories used as search terms for web-scraping (n = 120).

| **Google Business Categories** | | | |
| --- | --- | --- | --- |
| Afghani Restaurant | Salad Shop | Ice Cream Shop | Deli |
| African Restaurant | Sandwich Shop | Indian Restaurant | Vegetarian Cafe and Deli |
| Alsace Restaurant | Convenience Store | Indonesian Restaurant | Chocolate Cafe |
| American Restaurant | Candy Store | Italian Restaurant | Cocktail Bar |
| An Hui Restaurant | Delivery service | Japanese Restaurant | Wine Bar |
| Anago Restaurant | Fast Food Restaurant | Juice Shop | Fish & Chips Restaurant |
| Andalusian Restaurant | Fish and Chips Takeaway | Kebab Shop | Fresh Food Market |
| Angler Fish Restaurant | Fried Chicken Takeaway | Korean Barbecue Restaurant | Frozen Yogurt Shop |
| Argentinian Restaurant | Gas station | Korean Restaurant | Fruit and Vegetable Store |
| Armenian Restaurant | Hamburger Restaurant | Lebanese Restaurant | Grocery Delivery Service |
| Asian Fusion Restaurant | Meal Takeaway | Liquor Store | Butcher Shop Deli |
| Asian Grocery Store | Pie Shop | Malaysian Restaurant | Cajun Restaurant |
| Asian Restaurant | Pizza Restaurant | Market | Cake Shop |
| Asturian Restaurant | Pizza Takeaway | Meal delivery | Restaurant |
| Australian Restaurant | Pastry Shop | Mexican Restaurant | Cafe |
| Austrian Restaurant | Bakery | Milk Delivery Service | Grocery Store |
| Authentic Japanese Restaurant | Bistro | Noodle Shop | Supermarket |
| Bagel Shop | Pub | Pasta Shop | Vietnamese Restaurant |
| Bangladeshi Restaurant | Bar | Pho Restaurant | Wine Store |
| Bar Restaurant Furniture Store | Bar & Grill | Pizza delivery | Wholesale Bakery |
| Barbecue Restaurant | Chicken Restaurant | Ramen Restaurant | Donut Shop |
| Basque Restaurant | Chinese Noodle Restaurant | Seafood Market | Dumpling Restaurant |
| Belgian Restaurant | Chinese Restaurant | Seafood Restaurant | Fine Dining Restaurant |
| Berry Restaurant | Chinese Takeaway | Shanghainese Restaurant | Butcher Shop |
| Brazilian Restaurant | Coffee Shop | Sichuan Restaurant |  |
| Breakfast Restaurant | Coffee Store | Singaporean Restaurant |  |
| British Restaurant | Cookie Shop | Spanish Restaurant |  |
| Brunch Restaurant | Cupcake Shop | Sri Lankan Restaurant |  |
| Bubble Tea | Delivery Chinese Restaurant | Sushi Restaurant |  |
| Buffet Restaurant | Dessert Restaurant | Taiwanese Restaurant |  |
| Bulgarian Restaurant | Dessert Shop | Thai Restaurant |  |
| Burmese Restaurant | Dim Sum Restaurant | Vegan Restaurant |  |
| Burrito Restaurant | Discount Supermarket | Vegetarian Restaurant |  |
